# 2-Nitro-*p*-phenyl­ene dibenzene­sulfonate

**DOI:** 10.1107/S1600536810002898

**Published:** 2010-01-30

**Authors:** Zongwei Yang

**Affiliations:** a Sichuan College of Chemical Technology, Luzhou 646005, People’s Republic of China

## Abstract

In the title compound, C_18_H_13_NO_8_S_2_, the nitro­phenyl ring forms dihedral angles of 46.67 (7) and 75.40 (6)° with the phenyl rings. The nitro group makes a dihedral angle of 26.13 (8)° with the attached ring. The crystal packing is stabilized by weak inter­molecular C—H⋯O hydrogen bonds.

## Related literature

For background to the use of phenolic esters in organic synthesis, see: Trollsås *et al.* (1996 ▶); Svensson *et al.* (1998 ▶); Atkinson *et al.* (2005 ▶); Hu *et al.* (2001 ▶). For a related structure, see: Ji *et al.* (2006 ▶). For bond-length data, see: Allen *et al.* (1987 ▶).
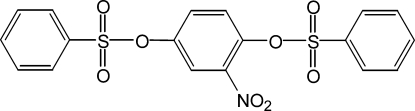


## Experimental

### 

#### Crystal data

C_18_H_13_NO_8_S_2_
*M* *_r_* = 435.43
Monoclinic, 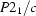

*a* = 11.669 (5) Å
*b* = 10.554 (4) Å
*c* = 15.343 (7) Å
β = 101.462 (7)°
*V* = 1851.9 (14) Å^3^
*Z* = 4
Mo *K*α radiation
μ = 0.34 mm^−1^
*T* = 113 K
0.30 × 0.07 × 0.06 mm

#### Data collection

Rigaku Saturn CCD area-detector diffractometer
Absorption correction: multi-scan (*CrystalClear*; Rigaku, 2007 ▶) *T*_min_ = 0.906, *T*_max_ = 0.980
15174 measured reflections
4386 independent reflections
3278 reflections with *I* > 2σ(*I*)
*R*_int_ = 0.035

#### Refinement

*R*[*F* ^2^ > 2σ(*F* ^2^)] = 0.042
*wR*(*F* ^2^) = 0.121
*S* = 1.08
4386 reflections
262 parameters
H-atom parameters constrained
Δρ_max_ = 1.11 e Å^−3^
Δρ_min_ = −0.50 e Å^−3^

### 

Data collection: *CrystalClear* (Rigaku, 2007 ▶); cell refinement: *CrystalClear*; data reduction: *CrystalClear*; program(s) used to solve structure: *SHELXS97* (Sheldrick, 2008 ▶); program(s) used to refine structure: *SHELXL97* (Sheldrick, 2008 ▶); molecular graphics: *SHELXTL* (Sheldrick, 2008 ▶); software used to prepare material for publication: *SHELXTL*.

## Supplementary Material

Crystal structure: contains datablocks global, I. DOI: 10.1107/S1600536810002898/bq2192sup1.cif

Structure factors: contains datablocks I. DOI: 10.1107/S1600536810002898/bq2192Isup2.hkl

Additional supplementary materials:  crystallographic information; 3D view; checkCIF report

## Figures and Tables

**Table 1:** Hydrogen-bond geometry (Å, °)

*D*—H⋯*A*	*D*—H	H⋯*A*	*D*⋯*A*	*D*—H⋯*A*
C3—H3⋯O4^i^	0.95	2.49	3.234 (3)	135
C9—H9⋯O8^ii^	0.95	2.46	3.368 (3)	160

Symmetry codes: (i) 
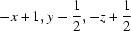
; (ii) 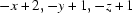.

**Fig. 1.**
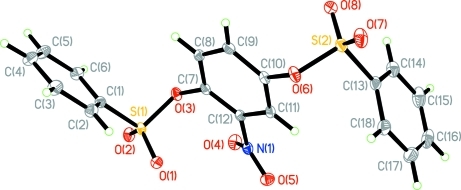
The molecular structure of the title compound, (I), with displacement ellipsoids drawn at 30% probability level.

**Fig. 2.**
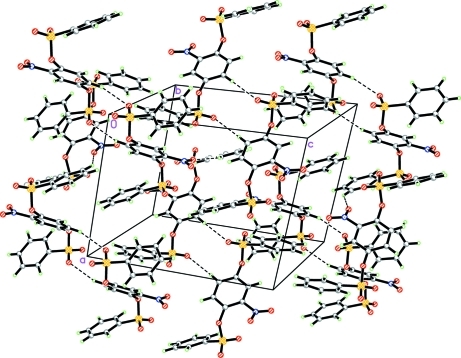
The crystal structure of (I), view along the a-axis. Dashed lines indicate C—H···O interactions.

## supplementary crystallographic information

### Comment

Phenolic esters can be used to synthesize some useful intermediates in organic synthesis (Trollsås *et al.*, 1996; Svensson *et al.*, 1998; Atkinson *et al.*, 2005; Hu *et al.*, 2001).

The compound (I) was prepared by the reaction of 2-nitrohydroquinone and 4-phenylsulfonyl chloride in the presence of triethylamine (Ji *et al.*, 2006) and its structure was reported here.

The molecular structure of (I) is shown in Fig. 1. The bond lengths and angles are within normal ranges (Allen *et al.*, 1987). The three aromatic rings ((C1 to C6), (C7 to C12) and (C13 to C18) form two dihedral angles of 46.67 (7)° and 75.40 (6)° in turn. The nitro group plane is connected with the aromatic plane with a dihedral angle of 26.13 (8)°. The torsion angles of C1—S1—O3—C7 and C10—O6—S2—C13 are 84.81 (15)° and -79.15 (16)°, respectively. In the crystal structure, intermolecular C—H···O hydrogen bonds link the molecules (Table 1, Figure 2).

### Experimental

2-nitrohydroquinone (78 mg, 0.5 mmol) was dissolved in chloroform (30 ml). To this solution, 4-phenylsulfonyl chloride (209 mg, 1.0 mmol) and triethylamine (101 mg, 1.0 mmol) were added and the reaction was stirred at room temperature for 3 h. The reaction mixture was extracted with dichloromethane and dried with anhydrous sodium sulphate. After concentration, the residue was separated by flash column chromatography and purified by recrystallization from chloroform (yield 156 mg, 72%, m.p. 393 K). Spectroscopic analysis: IR (KBr, ν, cm^-1^): 3075, 1541, 1483, 1379, 1198, 1165, 1091, 855, 733.

### Refinement

All H atoms were positioned geometrically and refined as riding (C—H = 0.95Å for aromatic H) and allowed to ride on their parent atoms, with U_iso_(H) =1.2U_eq_(parent).

### Crystal data

**Table:**

C_18_H_13_NO_8_S_2_	*F*(000) = 896
*M**_r_* = 435.43	*D*_x_ = 1.562 Mg m^−^^3^
Monoclinic, *P*2_1_/*c*	Mo *K*α radiation, λ = 0.71073 Å
Hall symbol: -P 2ybc	Cell parameters from 6872 reflections
*a* = 11.669 (5) Å	θ = 1.4–27.9°
*b* = 10.554 (4) Å	µ = 0.34 mm^−^^1^
*c* = 15.343 (7) Å	*T* = 113 K
β = 101.462 (7)°	Prism, colorless
*V* = 1851.9 (14) Å^3^	0.30 × 0.07 × 0.06 mm
*Z* = 4	

### Data collection

**Table:**

Rigaku Saturn CCD area-detector diffractometer	4386 independent reflections
Radiation source: Rotating anode	3278 reflections with *I* > 2σ(*I*)
multilayer	*R*_int_ = 0.035
Detector resolution: 14.63 pixels mm^-1^	θ_max_ = 27.8°, θ_min_ = 1.8°
ω and φ scans	*h* = −15→15
Absorption correction: multi-scan (*CrystalClear*; Rigaku, 2007)	*k* = −12→13
*T*_min_ = 0.906, *T*_max_ = 0.980	*l* = −20→14
15174 measured reflections	

### Refinement

**Table:**

Refinement on *F*^2^	Primary atom site location: structure-invariant direct methods
Least-squares matrix: full	Secondary atom site location: difference Fourier map
*R*[*F*^2^ > 2σ(*F*^2^)] = 0.042	Hydrogen site location: inferred from neighbouring sites
*wR*(*F*^2^) = 0.121	H-atom parameters constrained
*S* = 1.08	*w* = 1/[σ^2^(*F*_o_^2^) + (0.0646*P*)^2^] where *P* = (*F*_o_^2^ + 2*F*_c_^2^)/3
4386 reflections	(Δ/σ)_max_ < 0.001
262 parameters	Δρ_max_ = 1.11 e Å^−^^3^
0 restraints	Δρ_min_ = −0.49 e Å^−^^3^

### Special details

**Table:**

Geometry. All esds (except the esd in the dihedral angle between two l.s. planes) are estimated using the full covariance matrix. The cell esds are taken into account individually in the estimation of esds in distances, angles and torsion angles; correlations between esds in cell parameters are only used when they are defined by crystal symmetry. An approximate (isotropic) treatment of cell esds is used for estimating esds involving l.s. planes.
Refinement. Refinement of *F*^2^ against ALL reflections. The weighted *R*-factor wR and goodness of fit *S* are based on *F*^2^, conventional *R*-factors *R* are based on *F*, with *F* set to zero for negative *F*^2^. The threshold expression of *F*^2^ > σ(*F*^2^) is used only for calculating *R*-factors(gt) etc. and is not relevant to the choice of reflections for refinement. *R*-factors based on *F*^2^ are statistically about twice as large as those based on *F*, and *R*- factors based on ALL data will be even larger.

### Fractional atomic coordinates and isotropic or equivalent isotropic displacement parameters (Å^2^)

**Table:**

	*x*	*y*	*z*	*U*_iso_*/*U*_eq_	
S1	0.47624 (5)	0.78506 (4)	0.24585 (3)	0.02226 (14)	
S2	1.02988 (5)	0.38180 (5)	0.34494 (3)	0.02633 (15)	
O1	0.46295 (13)	0.67436 (12)	0.19112 (9)	0.0252 (3)	
O2	0.43170 (13)	0.90467 (13)	0.21175 (10)	0.0290 (4)	
O3	0.61466 (13)	0.81300 (12)	0.27823 (10)	0.0248 (3)	
O4	0.67578 (15)	0.81516 (13)	0.11784 (10)	0.0337 (4)	
O5	0.69050 (14)	0.62624 (13)	0.06595 (9)	0.0302 (4)	
O6	0.88958 (13)	0.37990 (13)	0.32927 (10)	0.0271 (3)	
O7	1.06055 (15)	0.25648 (14)	0.37645 (10)	0.0369 (4)	
O8	1.07180 (14)	0.49022 (15)	0.39729 (10)	0.0346 (4)	
N1	0.69459 (15)	0.70129 (15)	0.12753 (11)	0.0233 (4)	
C1	0.42957 (18)	0.75292 (18)	0.34514 (13)	0.0224 (4)	
C2	0.4055 (2)	0.62808 (19)	0.36603 (14)	0.0281 (5)	
H2	0.4156	0.5599	0.3278	0.034*	
C3	0.3661 (2)	0.6070 (2)	0.44472 (15)	0.0341 (6)	
H3	0.3498	0.5230	0.4610	0.041*	
C4	0.3503 (2)	0.7068 (2)	0.49945 (15)	0.0334 (5)	
H4	0.3208	0.6913	0.5519	0.040*	
C5	0.3772 (2)	0.8296 (2)	0.47808 (15)	0.0336 (6)	
H5	0.3686	0.8973	0.5171	0.040*	
C6	0.4165 (2)	0.85408 (19)	0.40072 (14)	0.0274 (5)	
H6	0.4343	0.9381	0.3856	0.033*	
C7	0.68888 (17)	0.70745 (17)	0.28997 (13)	0.0212 (4)	
C8	0.72464 (18)	0.66007 (18)	0.37471 (14)	0.0236 (4)	
H8	0.7001	0.6999	0.4235	0.028*	
C9	0.79652 (18)	0.55410 (19)	0.38883 (14)	0.0242 (4)	
H9	0.8229	0.5218	0.4472	0.029*	
C10	0.82902 (17)	0.49626 (18)	0.31632 (13)	0.0218 (4)	
C11	0.79618 (17)	0.54331 (17)	0.23077 (13)	0.0212 (4)	
H11	0.8203	0.5032	0.1820	0.025*	
C12	0.72676 (17)	0.65101 (17)	0.21879 (13)	0.0199 (4)	
C13	1.05739 (19)	0.40052 (19)	0.23713 (13)	0.0255 (5)	
C14	1.1317 (2)	0.4954 (2)	0.22087 (16)	0.0348 (5)	
H14	1.1657	0.5524	0.2667	0.042*	
C15	1.1561 (2)	0.5059 (2)	0.13575 (18)	0.0460 (7)	
H15	1.2078	0.5700	0.1233	0.055*	
C16	1.1054 (3)	0.4237 (3)	0.06989 (16)	0.0470 (7)	
H16	1.1222	0.4314	0.0120	0.056*	
C17	1.0300 (3)	0.3298 (3)	0.08698 (16)	0.0451 (7)	
H17	0.9949	0.2740	0.0407	0.054*	
C18	1.0057 (2)	0.3169 (2)	0.17063 (16)	0.0352 (6)	
H18	0.9547	0.2521	0.1828	0.042*	

### Atomic displacement parameters (Å^2^)

**Table:**

	*U*^11^	*U*^22^	*U*^33^	*U*^12^	*U*^13^	*U*^23^
S1	0.0194 (3)	0.0219 (3)	0.0260 (3)	0.00342 (19)	0.0056 (2)	0.0013 (2)
S2	0.0199 (3)	0.0352 (3)	0.0239 (3)	0.0062 (2)	0.0046 (2)	0.0068 (2)
O1	0.0246 (8)	0.0264 (7)	0.0247 (7)	0.0009 (6)	0.0049 (6)	−0.0019 (6)
O2	0.0293 (9)	0.0253 (7)	0.0337 (8)	0.0094 (6)	0.0097 (7)	0.0064 (6)
O3	0.0191 (8)	0.0197 (7)	0.0358 (8)	0.0021 (5)	0.0063 (6)	−0.0014 (6)
O4	0.0399 (10)	0.0241 (8)	0.0382 (9)	0.0079 (7)	0.0102 (8)	0.0108 (7)
O5	0.0348 (9)	0.0310 (8)	0.0246 (8)	−0.0030 (7)	0.0055 (7)	0.0013 (7)
O6	0.0192 (8)	0.0278 (7)	0.0350 (8)	0.0049 (6)	0.0069 (7)	0.0087 (6)
O7	0.0337 (10)	0.0424 (9)	0.0373 (9)	0.0174 (7)	0.0132 (8)	0.0187 (8)
O8	0.0247 (9)	0.0511 (9)	0.0268 (8)	0.0040 (7)	0.0023 (7)	−0.0051 (7)
N1	0.0200 (9)	0.0249 (9)	0.0261 (9)	0.0002 (7)	0.0071 (7)	0.0038 (7)
C1	0.0191 (10)	0.0236 (10)	0.0241 (10)	0.0014 (8)	0.0032 (8)	−0.0007 (8)
C2	0.0318 (13)	0.0228 (10)	0.0309 (11)	−0.0029 (9)	0.0093 (10)	−0.0063 (9)
C3	0.0443 (15)	0.0274 (11)	0.0326 (12)	−0.0097 (10)	0.0125 (11)	−0.0016 (9)
C4	0.0452 (15)	0.0326 (12)	0.0237 (11)	−0.0062 (10)	0.0100 (10)	−0.0024 (9)
C5	0.0461 (16)	0.0278 (11)	0.0274 (11)	0.0003 (10)	0.0090 (11)	−0.0064 (9)
C6	0.0328 (13)	0.0203 (10)	0.0287 (11)	0.0022 (8)	0.0053 (10)	−0.0005 (8)
C7	0.0156 (10)	0.0193 (9)	0.0284 (10)	−0.0013 (7)	0.0038 (8)	−0.0018 (8)
C8	0.0180 (10)	0.0277 (10)	0.0262 (10)	−0.0040 (8)	0.0069 (8)	−0.0051 (9)
C9	0.0193 (10)	0.0292 (10)	0.0230 (10)	−0.0031 (8)	0.0017 (8)	0.0026 (9)
C10	0.0155 (10)	0.0227 (9)	0.0272 (10)	0.0012 (8)	0.0046 (8)	0.0042 (8)
C11	0.0179 (10)	0.0234 (10)	0.0237 (10)	−0.0007 (8)	0.0074 (8)	0.0001 (8)
C12	0.0165 (10)	0.0201 (9)	0.0230 (10)	−0.0014 (7)	0.0040 (8)	0.0030 (8)
C13	0.0232 (11)	0.0318 (11)	0.0223 (10)	0.0102 (9)	0.0061 (8)	0.0052 (9)
C14	0.0304 (13)	0.0365 (12)	0.0386 (13)	0.0070 (10)	0.0097 (10)	0.0058 (10)
C15	0.0442 (16)	0.0480 (15)	0.0527 (16)	0.0152 (12)	0.0265 (13)	0.0218 (13)
C16	0.0529 (18)	0.0647 (17)	0.0274 (12)	0.0351 (15)	0.0179 (12)	0.0162 (13)
C17	0.0471 (17)	0.0592 (16)	0.0262 (12)	0.0264 (14)	0.0002 (12)	−0.0033 (12)
C18	0.0320 (13)	0.0384 (12)	0.0350 (12)	0.0117 (10)	0.0061 (10)	−0.0024 (10)

### Geometric parameters (Å, °)

**Table:**

S1—O2	1.4246 (14)	C5—H5	0.9500
S1—O1	1.4291 (15)	C6—H6	0.9500
S1—O3	1.6198 (16)	C7—C8	1.378 (3)
S1—C1	1.750 (2)	C7—C12	1.391 (3)
S2—O8	1.4274 (16)	C8—C9	1.389 (3)
S2—O7	1.4291 (15)	C8—H8	0.9500
S2—O6	1.6069 (17)	C9—C10	1.386 (3)
S2—C13	1.758 (2)	C9—H9	0.9500
O3—C7	1.401 (2)	C10—C11	1.384 (3)
O4—N1	1.225 (2)	C11—C12	1.387 (3)
O5—N1	1.226 (2)	C11—H11	0.9500
O6—C10	1.411 (2)	C13—C14	1.380 (3)
N1—C12	1.474 (2)	C13—C18	1.393 (3)
C1—C6	1.394 (3)	C14—C15	1.395 (3)
C1—C2	1.398 (3)	C14—H14	0.9500
C2—C3	1.393 (3)	C15—C16	1.373 (4)
C2—H2	0.9500	C15—H15	0.9500
C3—C4	1.383 (3)	C16—C17	1.385 (4)
C3—H3	0.9500	C16—H16	0.9500
C4—C5	1.388 (3)	C17—C18	1.375 (4)
C4—H4	0.9500	C17—H17	0.9500
C5—C6	1.379 (3)	C18—H18	0.9500
			
O2—S1—O1	121.30 (9)	C8—C7—O3	118.26 (18)
O2—S1—O3	102.71 (8)	C12—C7—O3	121.64 (17)
O1—S1—O3	108.31 (8)	C7—C8—C9	120.0 (2)
O2—S1—C1	109.73 (9)	C7—C8—H8	120.0
O1—S1—C1	109.68 (9)	C9—C8—H8	120.0
O3—S1—C1	103.42 (9)	C10—C9—C8	118.85 (19)
O8—S2—O7	121.12 (10)	C10—C9—H9	120.6
O8—S2—O6	108.61 (9)	C8—C9—H9	120.6
O7—S2—O6	102.61 (9)	C11—C10—C9	122.31 (19)
O8—S2—C13	109.46 (10)	C11—C10—O6	118.83 (18)
O7—S2—C13	110.01 (10)	C9—C10—O6	118.65 (17)
O6—S2—C13	103.41 (9)	C10—C11—C12	117.65 (19)
C7—O3—S1	116.62 (12)	C10—C11—H11	121.2
C10—O6—S2	118.49 (12)	C12—C11—H11	121.2
O4—N1—O5	124.14 (17)	C11—C12—C7	121.03 (18)
O4—N1—C12	118.28 (17)	C11—C12—N1	116.91 (18)
O5—N1—C12	117.56 (16)	C7—C12—N1	122.06 (17)
C6—C1—C2	122.0 (2)	C14—C13—C18	121.6 (2)
C6—C1—S1	118.36 (16)	C14—C13—S2	119.40 (17)
C2—C1—S1	119.60 (16)	C18—C13—S2	118.99 (18)
C3—C2—C1	117.77 (19)	C13—C14—C15	118.7 (2)
C3—C2—H2	121.1	C13—C14—H14	120.7
C1—C2—H2	121.1	C15—C14—H14	120.7
C4—C3—C2	120.8 (2)	C16—C15—C14	120.0 (3)
C4—C3—H3	119.6	C16—C15—H15	120.0
C2—C3—H3	119.6	C14—C15—H15	120.0
C3—C4—C5	120.3 (2)	C15—C16—C17	120.7 (2)
C3—C4—H4	119.9	C15—C16—H16	119.7
C5—C4—H4	119.9	C17—C16—H16	119.7
C6—C5—C4	120.5 (2)	C18—C17—C16	120.3 (2)
C6—C5—H5	119.7	C18—C17—H17	119.9
C4—C5—H5	119.7	C16—C17—H17	119.9
C5—C6—C1	118.6 (2)	C17—C18—C13	118.8 (2)
C5—C6—H6	120.7	C17—C18—H18	120.6
C1—C6—H6	120.7	C13—C18—H18	120.6
C8—C7—C12	120.10 (18)		
			
O2—S1—O3—C7	−161.01 (14)	S2—O6—C10—C9	−95.9 (2)
O1—S1—O3—C7	−31.54 (16)	C9—C10—C11—C12	−1.1 (3)
C1—S1—O3—C7	84.81 (15)	O6—C10—C11—C12	173.68 (17)
O8—S2—O6—C10	37.06 (17)	C10—C11—C12—C7	−1.8 (3)
O7—S2—O6—C10	166.41 (14)	C10—C11—C12—N1	178.80 (17)
C13—S2—O6—C10	−79.15 (16)	C8—C7—C12—C11	3.0 (3)
O2—S1—C1—C6	−33.48 (19)	O3—C7—C12—C11	−177.22 (17)
O1—S1—C1—C6	−169.09 (16)	C8—C7—C12—N1	−177.57 (18)
O3—S1—C1—C6	75.54 (18)	O3—C7—C12—N1	2.2 (3)
O2—S1—C1—C2	145.86 (17)	O4—N1—C12—C11	−151.69 (18)
O1—S1—C1—C2	10.3 (2)	O5—N1—C12—C11	27.0 (3)
O3—S1—C1—C2	−105.11 (17)	O4—N1—C12—C7	28.9 (3)
C6—C1—C2—C3	0.7 (3)	O5—N1—C12—C7	−152.42 (19)
S1—C1—C2—C3	−178.60 (17)	O8—S2—C13—C14	12.8 (2)
C1—C2—C3—C4	0.7 (3)	O7—S2—C13—C14	−122.56 (18)
C2—C3—C4—C5	−2.1 (4)	O6—S2—C13—C14	128.44 (17)
C3—C4—C5—C6	2.1 (4)	O8—S2—C13—C18	−168.81 (16)
C4—C5—C6—C1	−0.6 (3)	O7—S2—C13—C18	55.8 (2)
C2—C1—C6—C5	−0.8 (3)	O6—S2—C13—C18	−53.20 (18)
S1—C1—C6—C5	178.57 (17)	C18—C13—C14—C15	−0.7 (3)
S1—O3—C7—C8	−97.86 (18)	S2—C13—C14—C15	177.62 (17)
S1—O3—C7—C12	82.4 (2)	C13—C14—C15—C16	0.7 (3)
C12—C7—C8—C9	−1.4 (3)	C14—C15—C16—C17	−0.1 (4)
O3—C7—C8—C9	178.82 (17)	C15—C16—C17—C18	−0.6 (4)
C7—C8—C9—C10	−1.3 (3)	C16—C17—C18—C13	0.6 (3)
C8—C9—C10—C11	2.6 (3)	C14—C13—C18—C17	0.0 (3)
C8—C9—C10—O6	−172.13 (17)	S2—C13—C18—C17	−178.28 (17)
S2—O6—C10—C11	89.2 (2)		

### Hydrogen-bond geometry (Å, °)

**Table:**

*D*—H···*A*	*D*—H	H···*A*	*D*···*A*	*D*—H···*A*
C3—H3···O4^i^	0.95	2.49	3.234 (3)	135
C9—H9···O8^ii^	0.95	2.46	3.368 (3)	160

Symmetry codes: (i) −*x*+1, *y*−1/2, −*z*+1/2; (ii) −*x*+2, −*y*+1, −*z*+1.
